# A chronology of landsliding based on archaeological and documentary data: Pavlovské vrchy Hills, Western Carpathian Flysch Belt

**DOI:** 10.1038/s41598-020-57551-4

**Published:** 2020-01-22

**Authors:** Michal Bíl, Oldřich Krejčí, Lukáš Dolák, Vladimíra Krejčí, Jan Martínek, Jiří Svoboda

**Affiliations:** 1 CDV – Transport Research Centre, Líšeňská 33a, 636 00 Brno, Czech Republic; 2 Czech Geological Survey, Klárov 3, 118 21 Prague, Czech Republic; 3 Faculty of Science, Masaryk University, Kotlářská 267/2, 611 37 Brno, Czech Republic; 4 Institute of Archaeology, Academy of Science of the Czech Republic, 69129 Dolní Věstonice, Czech Republic

**Keywords:** Natural hazards, Geomorphology

## Abstract

Pavlovské vrchy Hills represent a distinctive elevation near the Czech-Austrian border where the active, dormant and relict landslides cover 12% of the area. Here we focused on the chronology of landsliding in this area using geological, archaeological and historical evidence. The earliest records of landsliding were determined in locations underlying the dated archaeological settlements. The Upper Paleolithic settlement complex dated between 37–24 ka cal BP, was originally deposited over these landslides. It was consequently destroyed in certain places by additional landslides preceding the last (Upper Pleniglacial) loess deposition (22 ka cal BP). These landslides took place before and after the Upper Paleolithic occupation of this area. This Pleistocene landslide event ranks among the oldest (albeit indirectly) dated landslide within the Czech part of the Western Carpathian Flysch Belt. The chronology of later, historical, landsliding was determined using written records (chronicles, official reports, archival evidence, etc.). Continuous records of landsliding were available as of the middle of the seventeenth century. The major concentration of landslides occurred at the beginning of the twentieth century (1910–1915). The 1663 landslide is currently the oldest landslide, in the Czech part of the Western Carpathian Flysch Belt, which was dated on the basis of documentary data.

## Introduction

The Western Carpathian Flysch Belt (WCFB) ranks among the areas most susceptible to landsliding in the Czech Republic^1–5^. As many as 15,215 landslides, representing 75% of all landslides registered within the entire Czech Republic (CR), can be found in the Czech part of the WCFB (6,465 km^2^). Holec *et al*.^6^ claimed that the ratio of landslide-affected areas in the entire WCFB is 13.6%. Certain local studies, conducted in the Czech part of WCFB, presented even higher numbers, up to 20%^7^.

Many types of landsliding can be found in the Czech WCFB, e.g. shallow landslides^3,8^, debris flows^9,10^, deep-seated landslides^2,11–13^, rockfalls^14–16^ or complex flow-like landslides^9,10,17^. A number of slopes in the highest parts of WCFB were also affected by DSGSD [e.g.^11,18,19^].

## Current Research on Landslide Dating in the Czech WCFB

### Pleistocene landslides

The vast majority of dated landsliding in the Czech part of WCFB are from the Holocene age. The inventory of the dated WCFB landslides with new radiocarbon dates (from woody and other organic remnants within the landslide body or from lakes on the landslide surface) allowed for the identification of landslide phases in the Holocene^20^. The results of dating the slope movements by the ^14^C determination method in the Czech WCFB have yielded so far results from the youngest phase of sliding in the Holocene^17^. Pánek *et al*.^17^ reported the results of dating from 13 localities ranging from 12.86–9.30 ka (Kykula locality; cal BP) to 510–470 years (Pluskovec locality; cal BP). Virtually, these data fall mostly into the Holocene period (0–11.734 ka cal BP).

Many well-known, large-scale landslides occurring in Czech WCFB still have not been dated, however. The primary cause of the lack of dating of these pre-Holocene landsliding here is the intensive erosion of the oldest landslide forms. Exceptions include the slopes of Kabátice in the Moravskoslezské Beskydy Mts. (around 51–56 ka cal BP with a continuation until <36 ka cal BP^21^); and the Malenik Ridge in the Moravian Gate^22^, where the Upper Pleistocene mass movements (47,704 ± 2346 cal b2k) were dated.

### Historical landslides

The general lack of suitable materials for landslide dating using geochronological methods, even among the youngest landslides, lead to utilization of other methods in WCFB, e.g. dendrochronological^16^. The existence of written documents (and related references to the current weather therein) has already been utilized by historical climatologists. Research focusing on landsliding which were active during historical times can also utilize this data. Špůrek^23^ provided the first comprehensive overview of dated landslide events with this method which covered the area of Central Europe, but also contained records from other parts of the world. The primary sources of his work were newspapers and historical documents. Several episodes of intensification of mass movements were identified in this work in WCFB (e.g.^23^). The most recent (1939, 1941, 1970, 1997, 2006 and 2010) were identified from the village of Halenkovice and its vicinity (approx. 70 km NE from Pavlovské vrchy Hills^7^);. An example of a landslide episode with a large spatial extent (e. g. the entire area of the CR and its vicinity) is that which took place in the spring of 1941^7,23–25^. Other historical documentation of landslide events, over more than the last 200 years, was conducted by Raška *et al*.^26,27^.

**Figure 1:**
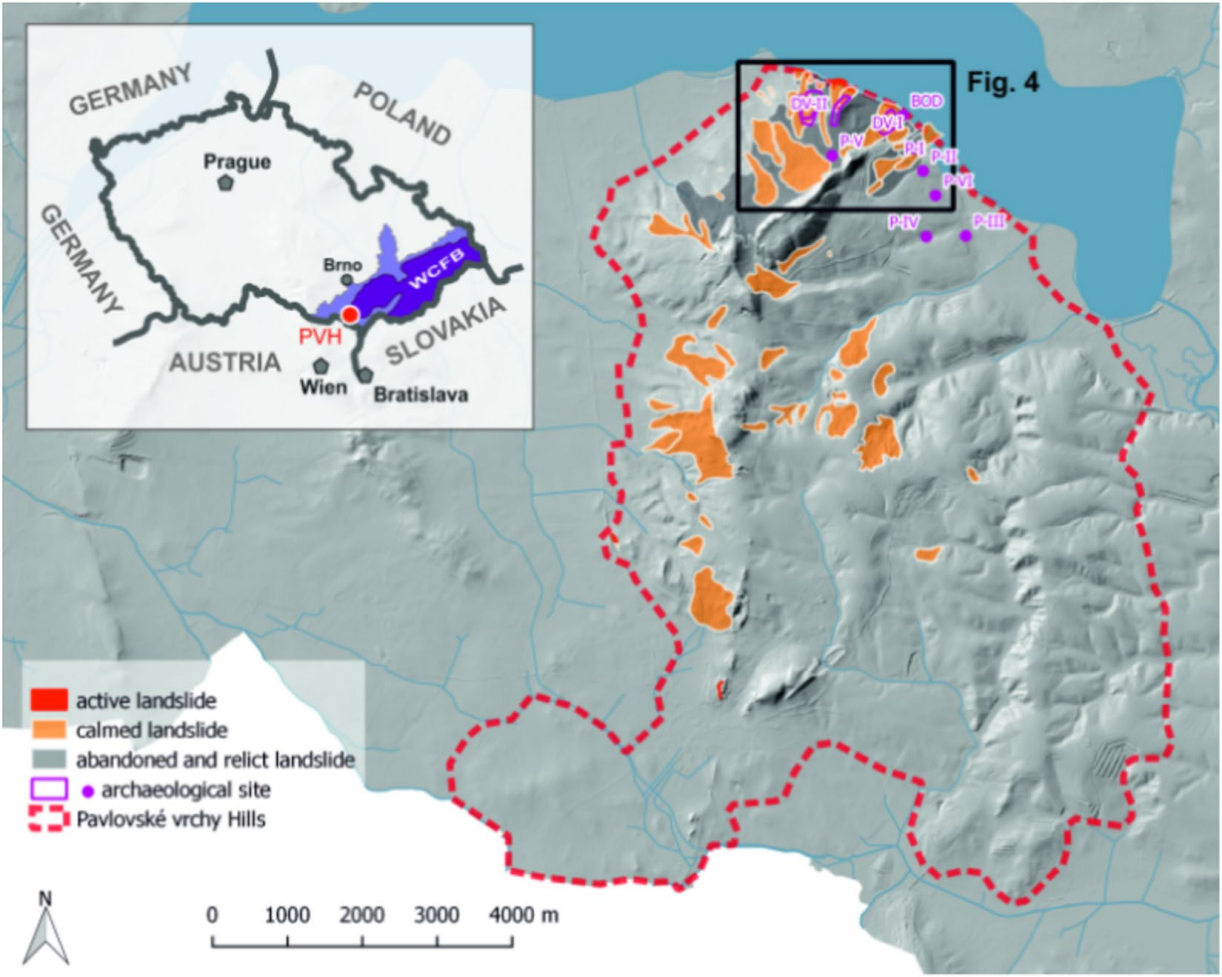
Pavlovské vrchy Hills (PVH) and its position within the Czech Republic and WCFB. Processed in QGIS Version 2.18.0 ‘Las Palmas’.

In this paper, which combines geological, archaeological, and historical records, we present evidence from the Pavlovské vrchy Hills for repeated landsliding which affected human settlements. We also synthetized data and built a chronology of landsliding for this part of the Czech WCFB from prehistory to the recent times. This area (located in Moravia, i.e., eastern part of CR; Fig. 1) has been systematically surveyed by archaeologists as of the 1920s. Landslides were mentioned a number of times along with the abundant archaeological findings^28–30^. It was also inhabited up until the end of World War II by a German population which carried out detailed reports, often mentioning natural processes, in their chronicles. Such systematic observations, including reports on landsliding, are not available for the rest of the Czech WCFB until the first quarter of the twentieth century.

### Geological and geomorphological setting

**Figure 2:**
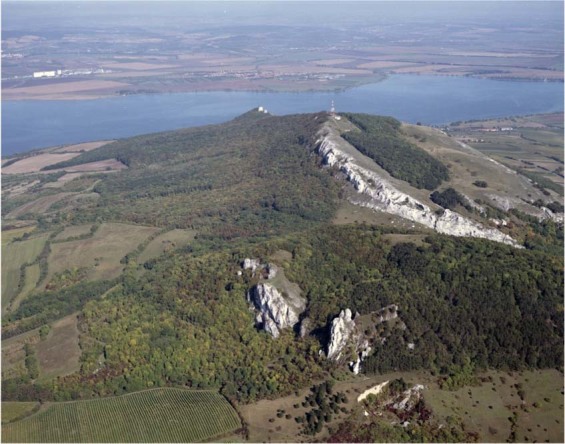
The Pavlovské vrchy Hills form a distinct dominant in a generally flat terrain which attracted Paleolithic humans for settlement. Photo Jan Vondra, Aerofot Brno.

**Figure 3:**
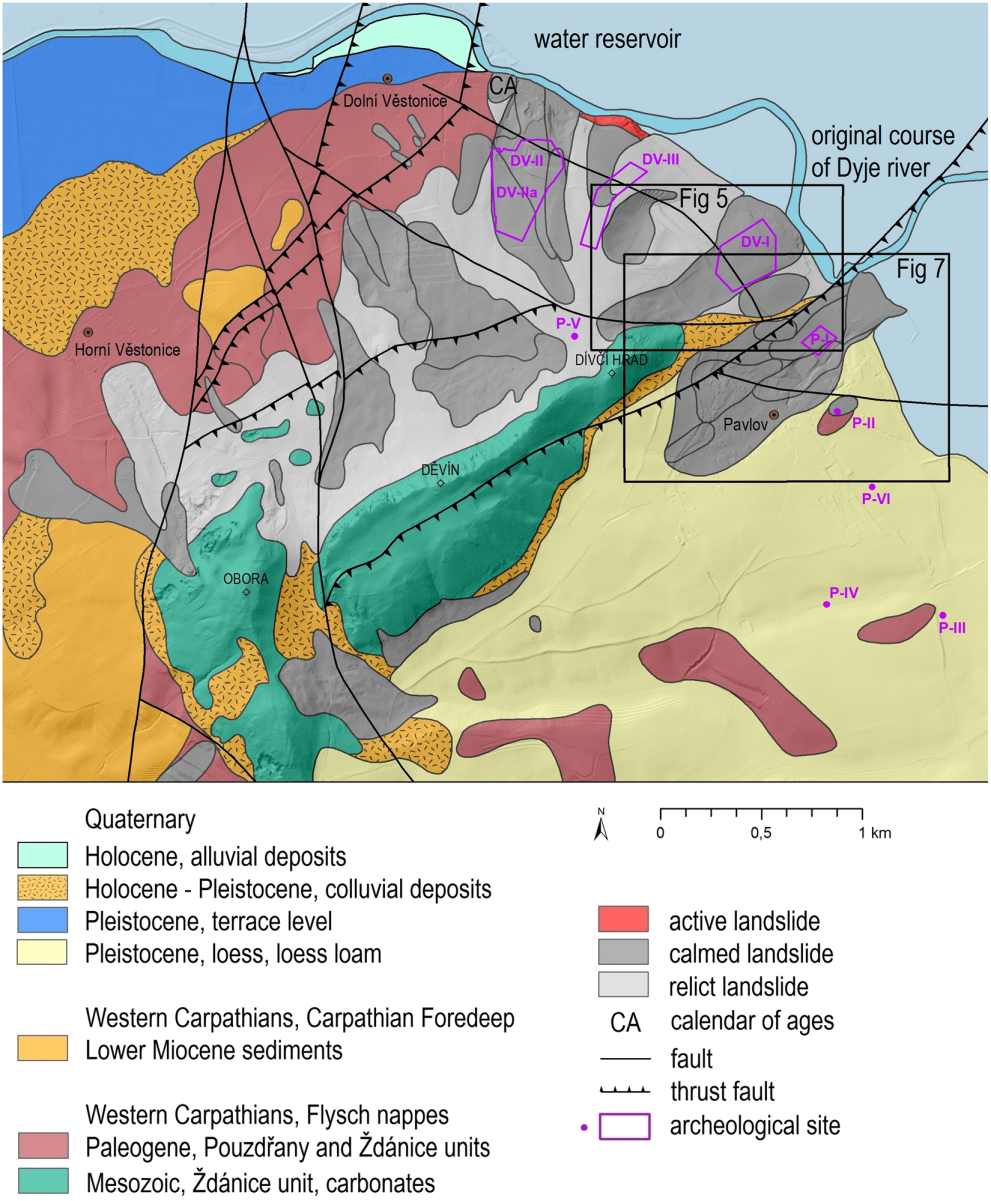
A geological map of the northern part of the Pavlovské vrchy Hills, which is bordered by a water reservoir to the north. Processed in ArcGIS 10.6.1 for desktop.

The topography of this area is strongly affected by the presence of relatively hard limestone which overhangs the neighboring weak flysch rocks forming a rather flat relief (Fig. 2). The Pavlovské vrchy Hills exceed the neighboring landscape up to 350 m^31^. The Hills are mainly built up by the rootless, tectonically transported, sediments of the lowermost unit of the WCFB nappe stack. They consist of numerous imbricates, duplexes, and partial thrust sheets of the Upper Jurassic to Lower Miocene strata. Tectonic klippen of Jurassic marls and carbonates and Upper Cretaceous (Turonian to Campanian) clastic rocks are tectonically incorporated into the thrust sheets of the younger, Paleogene to Lower Miocene sequences (Fig. 3).

The Pavlovské vrchy Hills make up part of the exterior Menilithic-Krosno Group of nappes of the WCFB. The area around Dolní Věstonice, built up of pre-Quaternary calcareous claystones, sandstones, and rare conglomerates, belongs to the Pouzdřany unit and mainly to the Waschberg – Ždánice units^32,33^. Waschberg – Ždánice units are the major frontal thrust system of the WCFB.

The area was the subject of erosion and denudation in the Late Miocene after the WCFB moved to its present position. Limestone bodies form an anticlinal nappe structure protruding from the surrounding, mostly claystone strata. It is a geological structure, typical for the Pavlovské vrchy Hills and disturbed by vertical fault tectonics, which resulted in the sliding of large block along the claystone surface. Pleistocene denudation and accumulation processes on the NW slopes of the Pavlovské vrchy Hills led to a complicated lithostratigraphic sequence, often disturbed by sliding. The block-stony rubble colluvium with the soil-clay matrix created large landslides that slid down the slopes. These landslides resulted in the next disintegration and a mixing of the material on the slopes arose and arises under appropriate climatic conditions, earthflows with devastating effects to both prehistoric settlements and modern villages.

Evidence of this process are the limestone blocks found in the riverbed of the Dyje River^28^. The role of bedrock and its structure in WCFB as a factor of landslide origin was investigated by Rybář^34^ who defined the typical case situations. The common pattern in the WCFB is the alteration of inclined rigid and plastic beds. It has been demonstrated that flysch slides commonly originate on slopes determined by downslope bedding.

The Quaternary cover of the Pavlovské vrchy Hills consists of fluvial sandy gravels of the Dyje River in several terraces (fluvial accumulations), colluvial slope sediments and aeolian sediments. During the warm interglacial and shorter interstadial periods of the Pleistocene, the fossil soils and their derivates (soil sediments) were formed in the aeolian sediments. Later, in the Holocene, the subfossil soils were developed in fluvial sandy loams (flood loam). This evidence illustrates the complicated geological evolution over the entire Quaternary in the last 2.588 Ma (www.stratigraphy.com; and^35,36^).

### Prehistoric and historical settlement of the pavlovské vrchy hills

#### Pleistocene

Although the earliest dated archaeological artifacts in the Pavlovské vrchy Hills belong to the Early Upper Paleolithic (probably Aurignacian), here we focus on the subsequent Middle Upper Paleolithic (Gravettian), the most extensive and the best dated archaeological unit.

Within the key regional section DV 09, found at the bottom of site DV II, (known as “brickyard” or “Calendar of Ages”) covering the complex sequence of Upper Pleistocene loess, paleosols, and colluvia, the archaeological layer was dated by C^14^ between 30,553–29,442 cal BP^37^. This layer also corresponds to an extensive Upper Paleolithic archaeological complex excavated over the last century at the Dolní Věstonice and Pavlov sites by Karel Absolon, Assien Bohmers, Bohuslav Klíma and Jiří Svoboda (see^28,38,39^).

These archaeological excavations provided 6 radiocarbon dates from the site of DV I^40^, 37 dates from DV II^41^, and 30 dates from Pavlov I^42^. The majority of these dates were measured at Groningen and Oxford Radiocarbon Laboratories and calibrated after OxCal v.4.2., using IntCal13 calibration curve^43^. These dates are also supported by a series of OSL dates from DV II^41,44^ and, most recently, by direct radiocarbon dates obtained from human fossils found at the same site and measured at the Aix-en-Provence Laboratory^45^. All these dates provide a consistent record of human occupation during the MIS3 period. In terms of microchronology, the dates place a scarce Early Upper Paleolithic (probably Aurignacian) occupation at several discrete spots at DV II and Pavlov I dated between 37–33 ka cal BP, followed by the dominant early Gravettian occupation at all sites between 33–29.5 ka cal BP, and by scarce evidence of the late Gravettian between 29.5–24 ka cal BP. At the sites of DV I, II, and Pavlov I, evidence of landsliding is documented both below and above the archaeological deposits. During the Last Glacial Maximum (a period culminating around 22 ka cal BP; see^46,47^), evidence of human settlements disappeared. The cultural layers and the evidence of landsliding were superposed by archaeologically sterile “Upper Pleniglacial Loess”^48^. The thickness of the Upper Loess layer in the Dolní Věstonice section DV 09 is about 5.8 m^37,49^. According to the optically stimulated luminescence (OSL) method of dating, the “Upper Pleniglacial Loess“ sedimentation in DV 09 took place between 27–20 ka cal BP^37,44^. This loess can be used for determining the age of the earlier slide movements that disturbed underlying Gravettian cultural layer.

#### Holocene

In contrast to the Pleistocene, evidence of the Holocene human settlement of the same area was continuous. It starts with the Mesolithic Period (11.0–7.5 ka cal BP). Neolithic farmers from the Middle East followed as of 8 ka BP^50^. After the Neolithic Period; about 7.5–6.3 ka BP and the Eneolithic Period (about 6.3–4.0 ka BP) the Pavlovské vrchy Hills became one of the most populated areas in Moravia^51^ in the Bronze Age (4.0–2.7 ka BP) and early Iron Age of the Hallstatt Period (2.7–2.4 ka BP). The La Tene (Celtic) Period inhabited this area for four centuries (about 2.4–2.0 ka BP) and during a short period (about 2.0 ka BP) was replaced by a German tribe of Suebi. It should be pointed out that a Roman military camp was located northwest of the Pavlovské vrchy Hills and dated to 1.8 ka BP^52^. At the beginning of the Migration Period (1.6 ka BP), most of the population of the Suebi tribe left and the Lombards inhabited the abandoned area. Already about 1.4 ka BP, however, the Lombards were replaced by Slavic people building several fortified settlements^53^. The area of the Pavlovské vrchy Hills went through a florescence about 1.1 ka BP as part of the powerful Great Moravia Empire. As of 0.7 ka BP, most of the current villages and towns existed, although some of them perished or were relocated over the following centuries. The main reasons for moving the villages, usually further from the rivers, was deforestation and more frequent floods^54^. At the same time, i.e. seven hundred years ago, the colonization of Germans to this area began and brought about an important German influence (language, skills, etc.). The number of documentary evidence increased since that time. German settlements stopped with the end of World War II in 1945^55^. The population density over history fluctuated depending on wars, famine or diseases (e.g. the Thirty Years’ War, 1618–1648).

## Data and Methods

### Documentary data

We used available historical data such as written records (chronicles, official reports, archival evidence etc.), newspapers, maps and photographs. This data was complemented by secondary sources (early scientific papers, the history of towns and villages, etc.). This kind of data allowed us to localize landsliding in terms of spatial and temporal accuracy. A number of documentary records were hand-written in Neo-Gothic italic script in German (in more recent times also in Czech). The terminology of landslides included a rich variety of terms: “Rutschung” [slide], “Erdrutschung” [landslide], “Erdrutsch” [sliding], “Einsturz” [slump] or “Senkung” [decline]. The information related to landslides was carefully transliterated and excerpted. Where possible, individual records were checked by cross-referencing to other evidence. Individual events, dates of occurrence (if they were available), the type of affected property, and the extent of damage and source of data became the basic information for further critical analyses and interpretations. The primary limitation when dating historical landslides in WCFB is the lack of available resources. Documentary data (chronicles, official reports, archival evidence, etc.) are only seldom available before the nineteenth century.

### LiDAR and digital elevation model data

**Figure 4:**
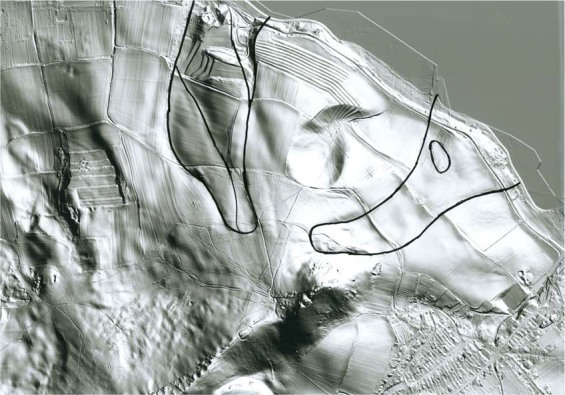
A detailed LiDAR picture where numerous tracks of landsliding can be identified. The conspicuous ridge in the central part is formed by Jurassic limestones and is a frequent source of landsliding and rockfalls.

Current geological maps, reports of geophysical investigation and actual LiDAR data (digital elevation model) were used in order to delineate the actual extent of landsliding in the Pavlovské vrchy Hills (Fig. 4). Slope deformations known in the study area are mainly typical landslides, earth or debris flows^56^ and vast, complex slope deformations up to many hundreds of metres in length^12,57^.

### Engineering-geological survey data

An engineering-geological survey related to the construction of Nové Mlýny water reservoirs was completed in 1967. These works^58^ provided data on the extent and depth of landslides related to the archaeological sites. Construction of the water reservoir took place between 1981 and 1988. The water in the reservoir has been in direct contact from that time with the frontal part of the slope instabilities (Fig. 3).

## Results

### Landslides identified on the basis of archaeological data

**Figure 5:**
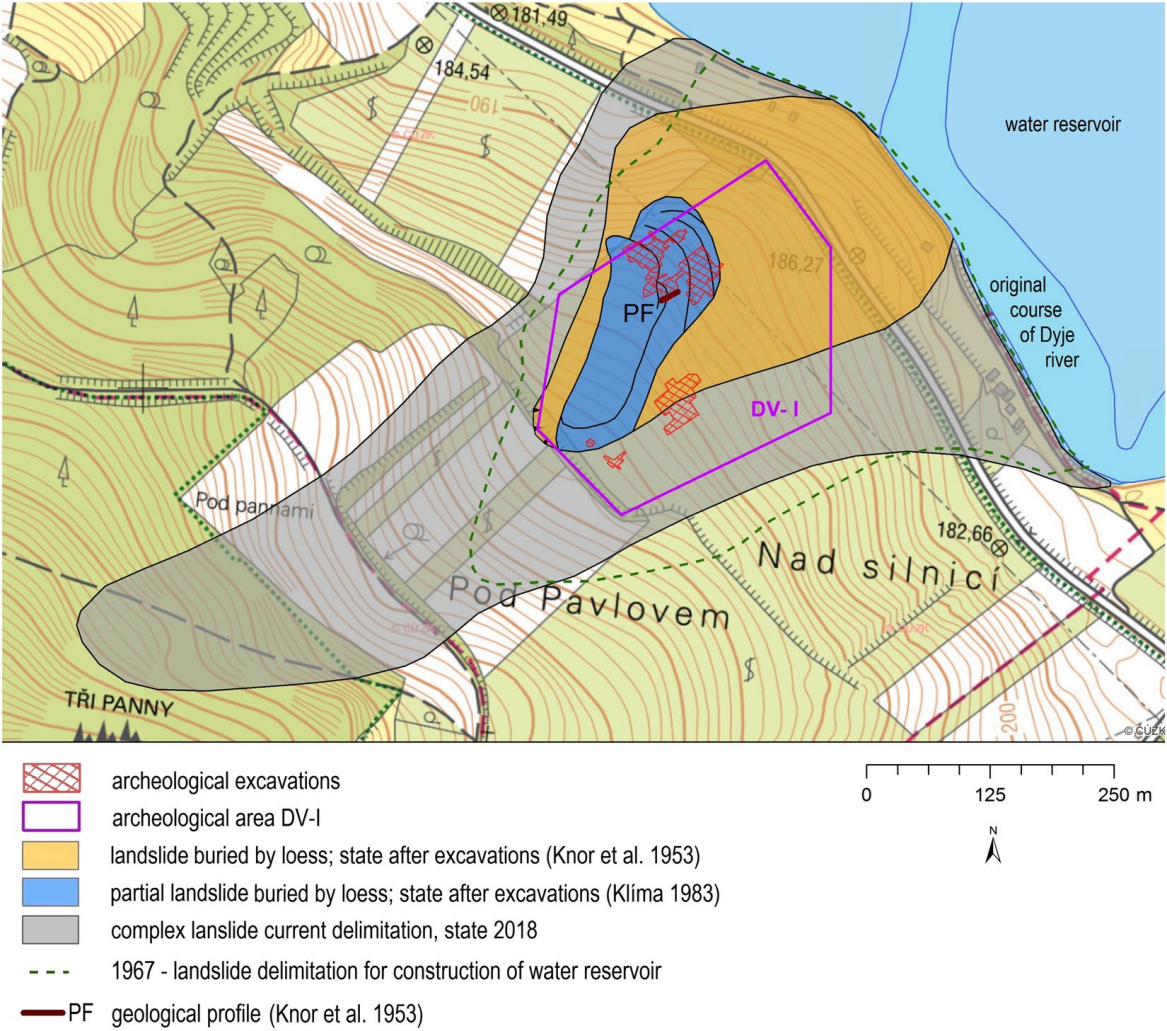
Landslide in the broader area of archaeological site DV I with a history of mapped boundary changes and dated findings. Processed in ArcGIS 10.6.1 for desktop.

An extensive complex landslide with the archaeological site DV I^28,29,38,40^ was a key locality for the reconstruction of the Upper Pleistocene slope movements. The development of mapping and knowledge about this landslide and individual archaeological excavations are shown in Fig. 5. The existence of several isolated settlements, located within the DV I archaeological site, determines that this area was inhabited and abandoned *several times*. Klíma^29^ suggested that this landslide area was selected to establish a Gravettian settlement as the wavy terrain included water springs, platforms suitable for building shelters, and a depression for bone and waste disposal.

**Figure 6:**
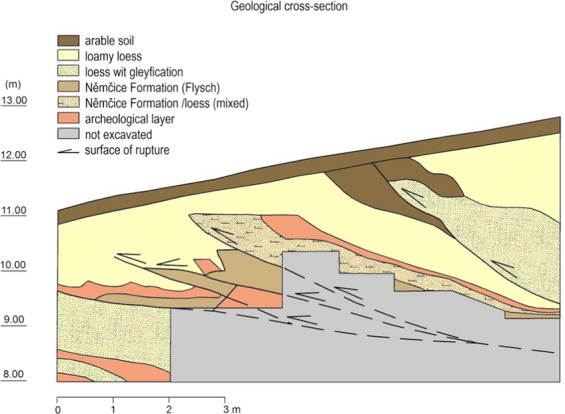
Excavation wall from 1947 with evidence of disturbance of the Gravettian archaeological layer due to slope deformations. Adapted after Knor *et al*.^28^.

Further local migrations of prehistoric hunters within the Pavlovské vrchy Hills could have been caused by partial activation of slope movements. Knor *et al*.^28^ have drawn valuable profiles through the partial walls of older archaeological excavations (already destroyed today), which unequivocally demonstrated the destruction of cultural layers by sliding movements (see Fig. 6). Moreover, in the same publication, it was stated that loess layers were stacked by sliding processes to 5–6 slices, which apparently caused superposition of 5 to 6 culture layers, one above another. This profile demonstrated that the slope movements that had affected the cultural layer were gradually completed during sedimentation of the *Upper Pleniglacial loess*. Determination of age of this *last loess deposition* was crucial for dating of the landsliding which affected and consequently destroyed Gravettian settlements. Excavation at the same site later yielded additional sections documenting a landsliding stage between the deposition of the cultural layer and the uppermost loess^40^.

The above-mentioned Upper Pleistocene landslides in all probability only rejuvenated much older rotationally-translational rupture zones. Flysch sediments of the Ždánice unit (WCFB) were transported in several phases almost to the surface along these sliding surfaces. These large landslides are therefore even older than the beginning of the Gravettian settlements.

### Landslides identified on the basis of documentary data

**Figure 7:**
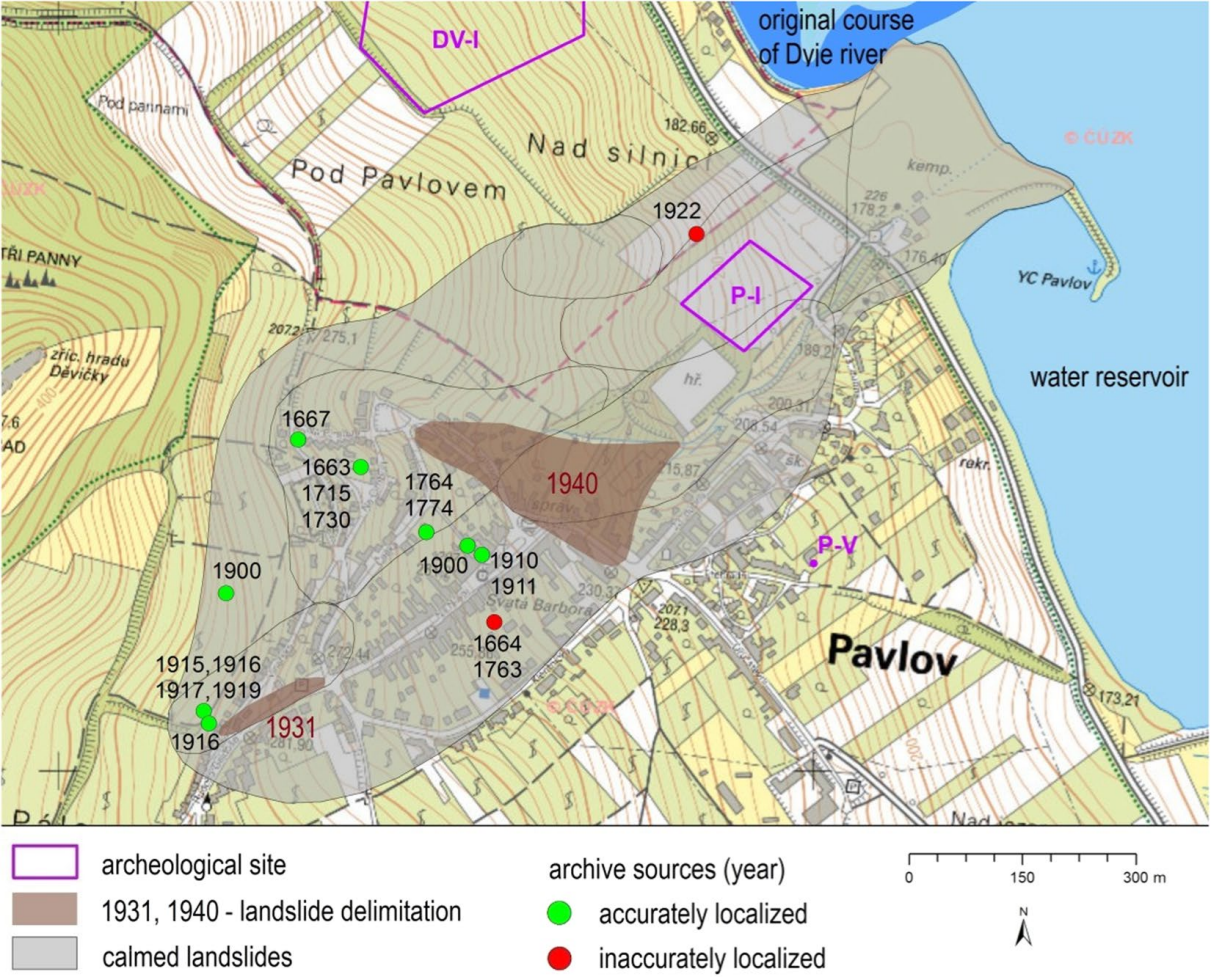
Pavlov village, the most frequently affected place by landsliding, at the Pavlovské vrchy Hills. Processed in ArcGIS 10.6.1 for desktop.

We found a written reference to the oldest landsliding in the Pavlovské vrchy Hills (Pavlov village in 1663; Fig. 7) in a book by Maca^59^ who compiled information on local life from the original German chronicles. This 1663 event thus presents the oldest record of landsliding based on the documentary data in WCFB in CR. There are only 3 older records, identified using the same technique, all located in the western part of CR. They took place during the sixteenth century^23^.

The database on landsliding (Table 1), which took place between 1663 and 1941, at Pavlovské vrchy Hills currently contains 30 events which occurred in 19 years in six villages. Many individual landslides were reactivated several times, but it was not always possible to distinguish reactivation from the emergence of new landsliding and identification of their precise positions was not always possible.

**Table 1:** Landslides at the Pavlovské vrchy Hills between 1663 and 1941. Dating based on documentary data.

Year	Month	Season	Municipality	Wet year*
1663	—	—	Pavlov	No
1667	—	—	Pavlov	No
1715	—	—	Pavlov	No
1730	—	—	Pavlov	No
1763	—	—	Pavlov	No
1770	—	—	Pavlov	Yes (Jun 1770)
1774	—	Spring	Pavlov	No
1830	—	Spring	Pavlov	No
1836	—	—	Perná	Yes
1900	—	Spring	Pavlov	locally yes
1900	—	Spring	Mikulov (Turold)	locally yes
1906	—	—	Dolní Věstonice	No
1910	—	Spring	Pavlov	Yes
1911	Jan	Winter	Klentnice	Yes (1910)
1911	Jan	Winter	Mikulov (Turold)	Yes (1910)
1911	Jan	Winter	Pavlov	Yes (1910)
1911	Jan	Winter	Perná	Yes (1910)
1911	Jan	Winter	Dolní Věstonice	Yes (1910)
1915	—	Autumn	Pavlov	Yes (Oct)
1915	—	Autumn	Klentnice	Yes (Oct)
1915	—	Autumn	Mikulov (Turold)	Yes (Oct)
1916	Jun	Spring	Pavlov	Yes (Jun)
1916	—	Spring	Mikulov (Turold)	Yes (Jun)
1917	—	—	Pavlov	No
1919	—	—	Pavlov	Yes (Nov)
1919	—	—	Klentnice	Yes (Nov)
1919**	—	Winter	Dolní Dunajovice	Yes (Nov)
1922	—	Spring	Dolní Věstonice	No
1922	—	Spring	Pavlov	No
1941	—	—	Pavlov	Yes

*Information about wet years derived from^66^. Data were further checked using the following works^67,68^ and information in local chronicles.

**Destruction of pseudokarst cave roofs on Liščí kopec hill.

#### Impacts of landsliding on local society

It was clear, from the available archaeological evidence, that the settlements of mammoth hunters were destroyed several times by landsliding. They therefore had to change their home for a number of times as also documented from archaeological data.

Concerning the historical landslides, they were not usually reported, if they did not cause any economic losses. As many as 30 landslides were identified between 1663 and 1941 in this area (Table 1). Pavlov village was the most frequently mentioned place (17-times) in term of landslide losses. Landslides also destroyed pseudokarst caves. This occurred in Dolní Dunajovice village in the winter of 1919–20 where a pseudokarst cave roof collapsed as a result of landsliding^60^.

Špůrek^23^ reported 15 cases of landsliding, with the oldest from 1906. Other landslides, as well as the previously mentioned by Špůrek^23^, were recorded by local documentary resources and early scientific works. The smallest number of reports about a landslide was one (1667, 1770 – Pavlov, 1900 – Mikulov, 1911 – Dolní Věstonice, 1919 – Dolní Dunajovice and 1941 – Pavlov), while other landslides were mentioned at least twice. Four references were available for landslides in 1774, 1900 and 1916. One landslide, which should have taken place in 1836 near Perná village^61^, can be clearly visible from a map from 3^rd^ Military survey which was prepared in the 1870s.

**Figure 8:**
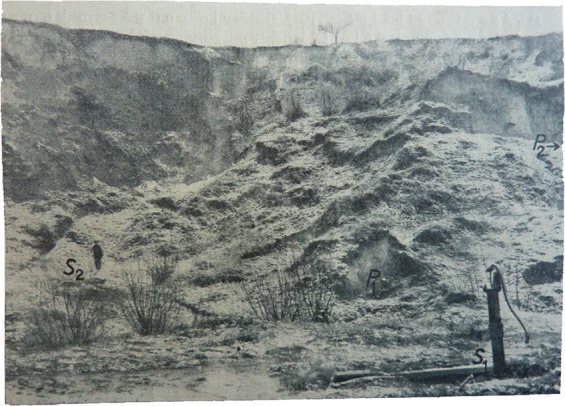
The 1906 landslide near Dolní Věstonice village. Photo from Woldřich and Stejskal^65^.

At least eight houses, ten farm buildings and seven cellars were completely destroyed over the 1663–1941 period. Another 17 buildings and 12 cellars were spoiled by landsliding. The oldest detailed description can be determined for spring 1774^59,62,63^. Other reports come from 1830^64^. Part of a clay quarry close to Dolní Věstonice was affected by landsliding in 1906^65^ (Fig. 8). Exceptionally wet weather in 1910 caused many landslides in January 1911. Two houses were completely destroyed in Pavlov in 1911 and many gardens deformed and moved up to 50 m downslope^63^. Another phase of landsliding took place between 1915 and 1917 when cracks were registered in almost all the buildings in Pavlov village, including the church and 15 houses and 11 cellars were destroyed^62^. The last intensification of landsliding was registered in 1941 when 3 cellars were destroyed, and many vineyards were damaged^59^. The above-mentioned landslide chronology can be compared to those previously published for WCFB^7^.

## Discussion

We presented the chronology of landsliding, for an area in the Czech WCFB, which encompasses the last five centuries. We also showed unique evidence of Pleistocene landsliding which took place after the termination of the Upper Paleolithic occupation. Finally, we demonstrated the earliest landsliding at the base of the Pleistocene sections that predate considerably the Upper Paleolithic period. No information on exact dates on landsliding between 22 ka cal BP and 1663 AD was available from this area.

The proven age of the active Pleistocene landsliding ranks among the oldest reactivations within the Czech WCFB. Despite the fact that some of the above-mentioned findings were already reported in older works^28,29^, no modern dating methods were available at that time to precisely date both quaternary sediments and remnants of bones. New approaches allowed for the identification of the above-mentioned findings as contemporary with the Gravettian settlements and the age of the Upper Pleniglacial loess which was deposited on the already destroyed Gravettian settlements. While some old landslides in arid and semi-arid regions of the world are still in morphologically distinctive forms, low resistance rocks to denudation together with humid climate of WCFB resulted that the oldest landslides can only be identified using other than direct morphological approaches, e.g. geophysical methods. The Upper Pleniglacial loess fortunately conserved the Gravettian settlements which could therefore be discovered in the twentieth century.

### Pleistocene chronology of landsliding

**Figure 9:**
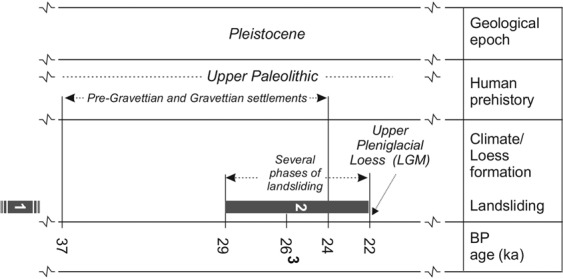
A Pleistocene chronology of landsliding at the Pavlovské vrchy Hills. 1 – the oldest landslides with unknown age, but older than 37 ka BP, which served as a basis for Pre-Gravettian and Gravettian settlements, 2 – Several phases of landsliding which affected the Gravettian settlements and caused their relocation within the PVH, 3 – an exactly dated landslide. LGM – Last Glacial Maximum.

A chronology of landsliding at the Pavlovské vrchy Hills during the Pleistocene can be reconstructed (see Fig. 9) as follows:

1. Basal large-scale landslides of an unknown age, formed by Jurassic limestone scree and redeposited Tertiary silts and marls. They created a versatile paleo-relief of elevations and side-gorges on the slopes of the Pavlovské vrchy Hills. The basal landslides are partially covered by loess and palaeosols.
2. These elevations and plateaus were consequently inhabited by Upper Palaeolithic (particularly Pre-Gravettian and consequently Gravettian) hunters, documented at several sites between 37–24 ka cal BP. The relevant stratigraphy demonstrated that basal landslides (no. 1 in Fig. 9) clearly predate the Upper Palaeolithic period. Details about these findings can be found at the following sites: DV I and II, Pavlov I^28,29,38,40,42^.
3. One of the cases, where a landslide of Tertiary silts and marl created a water-fan, subsequently filled by water molluscs and mammoth bones, was documented in a gorge below the DV II site. The dating and archaeological context are consistent with the large series of dates from the adjacent settlement. A charcoal sample taken from the location of DV II provided a date of 26,100 ± 200 cal BP^30^ which can be used for identification of the age of the landsliding.
4. Local landslides within the Gravettian settlements. These events partially destroyed the Gravettian layers and were subsequently covered by later Upper Pleniglacial loess sedimentation. These landslides cannot be dated precisely because they only disintegrated the already deposited cultural layer. They occurred between the end of the settlement and the formation of the cultural beds and the end of the sedimentation of Upper Pleniglacial loess. Related site DV I was described in^38,42^.

### Historical records and landsliding

Despite the importance of documentary data for the study of landslides in the Pavlovské vrchy Hills, several uncertainties have to be considered. Some of the original chronicles and other narrative sources were carried away by their authors or their offspring after 1945. A great deal of documentary data was also damaged (both deliberately and unintentional) over the centuries (wars, natural disaster, inappropriate storing, etc.) and their references are known only via early scientific papers and the histories of towns and villages. The landslides usually did not attract the attention of chroniclers if they did not cause any economic losses. Most of the reports described the occurrence of landslides only briefly and more detailed accounts appeared as of the second half of the eighteenth century. The oldest accounts therefore made the exact localization of landslides impossible in some cases and the same uncertainty was related to the determination of the exact date when the landslide occurred. More recent documentary data did not have to be more accurate, however, than the older one and had to be checked carefully. Finally, except for the different informational value of documentary data, the repeating damages at the same localities and same buildings, which were continuously repaired, made it more difficult to determine the accurate extent of the damage. Despite all these uncertainties, documentary evidence is valuable and an indispensable source of data describing the occurrence as well as the consequences of landslides in the Pavlovské vrchy Hills during the last four centuries. The last stage of the sliding activity in the Pavlovské vrchy Hills was documented in 2014, when there was a great deal of damage to the road infrastructure. The cause of this activity was the high rainfall value in September 2014. Engineering-geological works provided data on the actual extent and depth of slope deformations (Fig. 3), but the activity was recorded outside archaeological sites. No landslides were recorded here between 1941 and 2014. The current state of landsliding in the area of Pavlovské vrchy Hills was identified using detailed field mapping and LIDAR data analysis.

## Conclusions

We studied the chronology of landsliding at the Pavlovské vrchy Hills (Czech part of the WCFB). Leaving aside the evidence of earlier landsliding in the subsoil, the findings about repeated landsliding, presented in this work, can be used as an example of landslide recurrence persisting from the Upper Pleistocene to the present. This work thereby provided new information about landsliding in the Czech WCFB. The main contribution of this work can be summarized as follows:

- We analyzed available geochronological data, related to the archaeological excavations, which helped us identify several previously unknown phases of landsliding from the Czech part of the WCFB.
- An indirect dating of landslides which destroyed the cultural Gravettian beds was further based on the known age at the base of the Upper Pleniglacial loess which conserved both the landslides and the settlements.
- The identification of the 1663 landslide in the village of Pavlov is thus the *oldest* landslide in Czech WCFB from the historical period, and the fourth oldest landslide in CR, dated using *documentary* data.
- Compilation of the chronology of landsliding (see Table 1) for the Pavlovské vrchy Hills between as the seventeenth century and information about related losses from landsliding.
